# Laparoscopic Colorectal Resection in Octogenarian Patients: Is it Safe? A Systematic Review and Meta-Analysis

**DOI:** 10.1097/MD.0000000000001765

**Published:** 2015-10-23

**Authors:** Minghao Xie, Huabo Qin, Qianxin Luo, Xiaosheng He, Ping Lan, Lei Lian

**Affiliations:** From the Department of Colorectal Surgery (MX, HQ, QL, XH, PL, LL); and Guangdong Provincial Key Laboratory of Colorectal and Pelvic Floor Diseases, the Sixth Affiliated Hospital, Sun Yat-sen University, Guangzhou, Guangdong, P.R. China (MX, HQ, QL, XH, PL, LL).

## Abstract

The population older than 80 years has been increasing. A significant proportion of colorectal diseases that require colorectal resection occur in very elderly patients. However, the benefits of laparoscopy remain controversial in octogenarians. A systematic review and meta-analysis of observational study was performed to compare clinical outcomes between laparoscopic versus open colorectal resection in octogenarians.

The PubMed, EMBASE, Ovid, Web of Science, and Cochrane databases from the years 1990 to 2015 were searched for studies that compare surgical outcomes between laparoscopic and open colorectal resection in octogenarians (≥80 years old).

Seven eligible studies including 528 laparoscopic and 484 open colorectal resections were identified. Laparoscopic approach was associated with lower rate of mortality (odds ratio [OR] 0.48, *P* = 0.03), overall complications (OR 0.54, *P* < 0.001), and prolonged ileus (OR 0.56, *P* = 0.009), quicker bowel function return (standardized mean difference [SMD] −0.50, *P* < 0.001), and shorter length of hospital stay (SMD −0.47, *P* = 0.007). No differences were found in anastomotic leak (OR 1.16, *P* = 0.72), respiratory complication (OR 0.60, *P* = 0.07), and reoperation (OR 0.85, *P* = 0.69).

Laparoscopic colorectal resection is as safe as open approach, and the short-term outcomes appear to be more favorable in octogenarians.

## INTRODUCTION

With the rapid improvement in health care and advances in modern medicine, global life expectancy increased dramatically over the past decade, and more people are expected to live to an advanced age. It is estimated that half of the individuals born today will live to the age of 100 years.^1^ In the United States, the population older than 80 years has increased from 9.19 million (3.26%) to 11.28 million (3.64%) over the past decade.^2^

Along with this is the increase in the number of very old patients. A significant proportion of colorectal diseases that require colorectal resection occur in this patient group. An article published in the *Lancet*^3^ showed that the incidence of postoperative morbidity and mortality increased progressively with age, and the overall survival reduced in the older patients, especially in the patients aged 65 years or older. This study also found that comorbidities, especially pulmonary diseases and cardiovascular problems in the older patients, were associated with higher incidence of postoperative morbidity and mortality.

Some randomized controlled trials have shown that laparoscopic colorectal surgery is safe and is associated with definite benefits in short-term surgical outcomes, such as shorter length of hospital stay, less postoperative pain, earlier recovery of bowel function, and more rapid return to regular activities, compared with conventional open surgery for selected adult patients.^4,5^ It is proposed that laparoscopic surgery may be the ideal option for the elderly patients.^6^ However, laparoscopic approach is associated with specific physiologic changes, which might influence the older patients adversely. For instance, the Trendelenburg positioning during laparoscopy and pneumoperitoneum may result in a significant reduction in stroke volume and cardiac outputs.^7^ These factors, together with existing comorbidities, have made surgeons reluctant to offer laparoscopy to the older patients.^8,9^ However, Chautard et al^10^ demonstrated, in a large case-matched study, that laparoscopic colorectal resection (LC) in older patients (>70 years) is similar to that in the younger patients (<70 years) in terms of early postoperative outcomes, despite significant higher incidence of cardiopulmonary comorbidities in the senior cohort. Therefore, we hypothesized that LC confers benefits over the open colorectal resection (OC) for the older population, particularly those who are aged 80 years or above.

The present meta-analysis was performed in an attempt to evaluate the safety and efficacy between LC versus OC in octogenarians.

## METHODS

### Literature Search

A literature search of the PubMed, EMBASE, Ovid, Web of Science, and Cochrane databases from the years 1990 to 2015 was performed to identify articles comparing laparoscopic versus open surgery for octogenarians undergoing colorectal resection for cancer or other diseases.

The following search terms and their combinations were used: (laparoscop^∗^ OR minimally invasive), (colectomy OR colorectal resection/surgery), (octogenarian^∗^ OR elderly OR older than 80). Both free text search and Mesh search headings for keywords were employed. The “related articles” function was used to broaden the search, and relevant articles referenced in the publications were also searched for additional studies for potential inclusion. No language restriction was used. The date of the most recent search was March 20, 2015.

### Study Selection

For inclusion in the meta-analysis, studies were required to fulfill the following criteria: clear definition of surgical approach (laparoscopic vs open colorectal surgery); appropriate age cut-off (aged 80 or above); concurrent controls included; adequate data to determine OR and confidence intervals (CIs); and no later publication of identical data.

Noncomparative studies, case series, and case reports were not included. Two authors (MX and HQ) independently assessed titles and abstracts of all identified studies and excluded those deemed irrelevant. Full-text articles of potentially relevant studies were obtained. Disagreements on inclusion were discussed and, if necessary, by involving an independent third author (LL).

### Outcome Measurements

Safety outcomes included total complication rate, wound infection, anastomotic leak, prolonged ileus, cardiovascular complication, respiratory complication, and mortality.

Surgical outcomes included bowel function recovery time, length of hospital stay, and perioperative reoperation.

### Quality and Bias Assessment

The quality of all the included studies was assessed by 2 authors (MX and HQ) using the modified Newcastle–Ottawa Scale.^11^ The quality of the studies was evaluated by examining 3 items: patient selection, comparability of LC and OC groups, and assessment of outcome. Graphical exploration with funnel plots was used to evaluate publication bias.^12^

### Statistical Analysis

Odds ratios were used to represent the likelihood of an adverse event in the LC group compared with that in the OC group. The ORs were combined using the Mantel–Haenszel method for the outcomes of interest.^13^ Statistical analyses for continuous variables were performed using the standardized mean difference (SMD). A negative SMD favored the laparoscopic surgery group. For all outcomes, the fixed and random-effect models were calculated. When *I*^2^ test rejected the assumption of study homogeneity (*I*^2^ value >50%), the fixed-effect model was considered inappropriate, and the random-effect model was adopted. For continuous variables such as medians with ranges, the mean and standard deviation were estimated as previously described by Hozo et al.^14^ All analyses were conducted using R (version 3.1.3, R Foundation for Statistical Computing, Vienna, Austria) with the “meta” package. A *P* value <0.05 was considered statistically significant.

### Ethics Approval

The study was reviewed and approved by the Institutional Review Board and the Ethics Committee of the Sixth Affiliated Hospital of Sun Yat-sen University, Guangzhou, China.

## RESULTS

**FIGURE 1:**
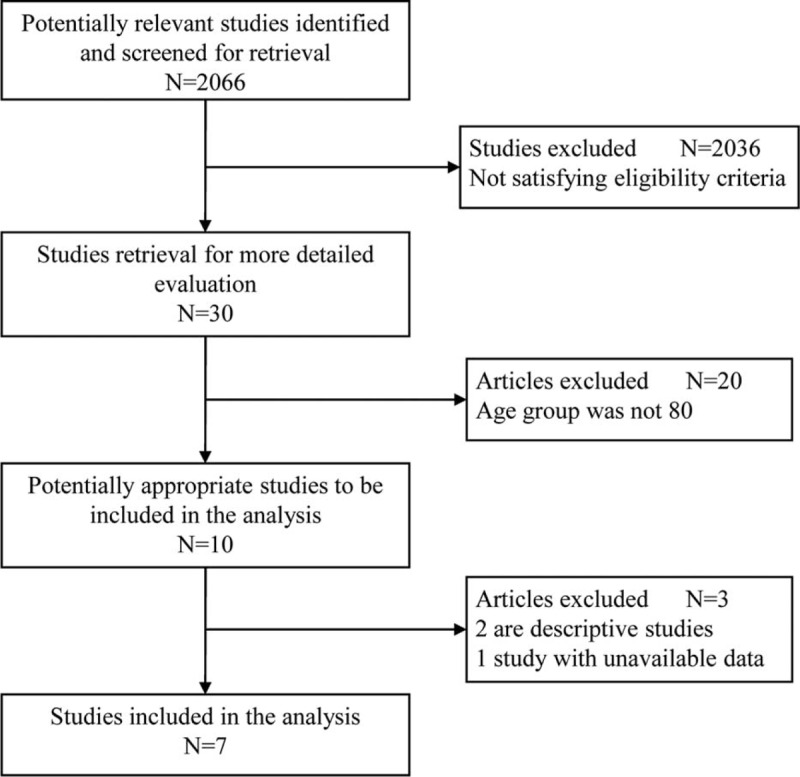
Flow chart of study selection process.

The inclusion and exclusion processes for identified articles are shown in Figure 1. Seven observational studies were finally included in the meta-analysis, comparing laparoscopic versus open surgery for octogenarians undergoing colorectal resection, using the predefined search strategy, containing a total of 1012 patients. There was no randomized controlled trial published. All the studies were single institutional. Sample size of the studies varied from 77 to 245 patients. Review of the data extraction showed 100% agreement between the 2 reviewers. There were 528 (52.2%) patients in the LC group and 484 (47.8%) patients in the OC group. The characteristics of the included studies are shown in Table 1. Surgery-related details are shown in Table 2. No significant differences were found between the 2 groups in indication for surgery or type of procedure in any of these studies.

**TABLE 1:**
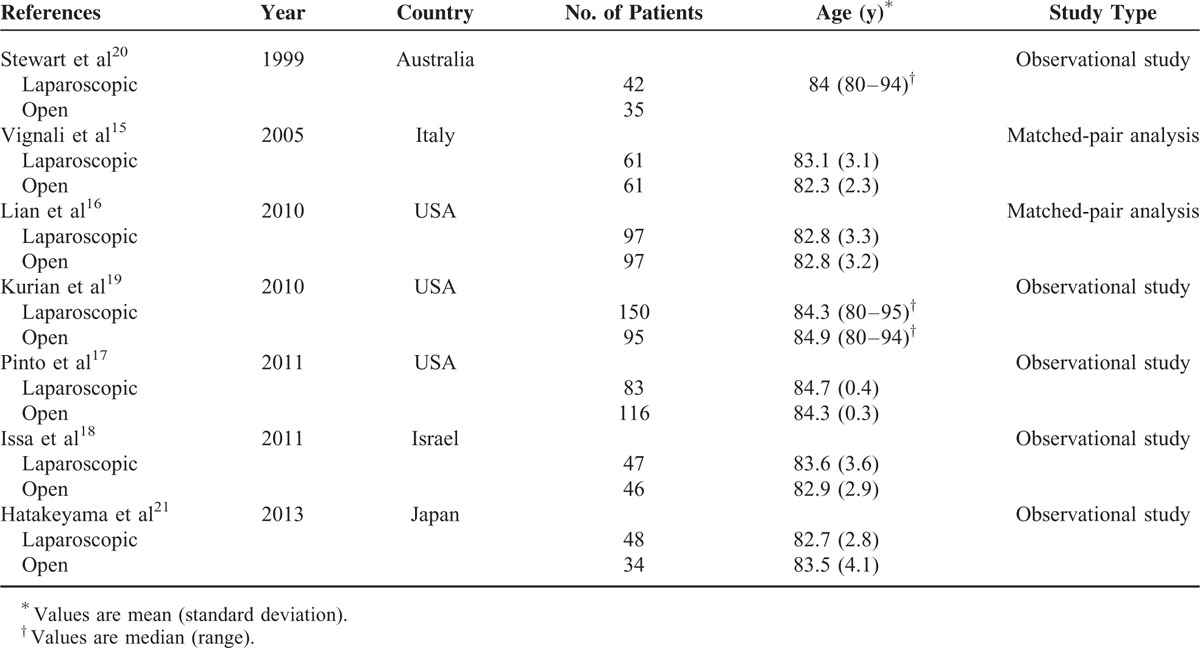
Characteristics of Included Controlled Studies

**TABLE 2:**
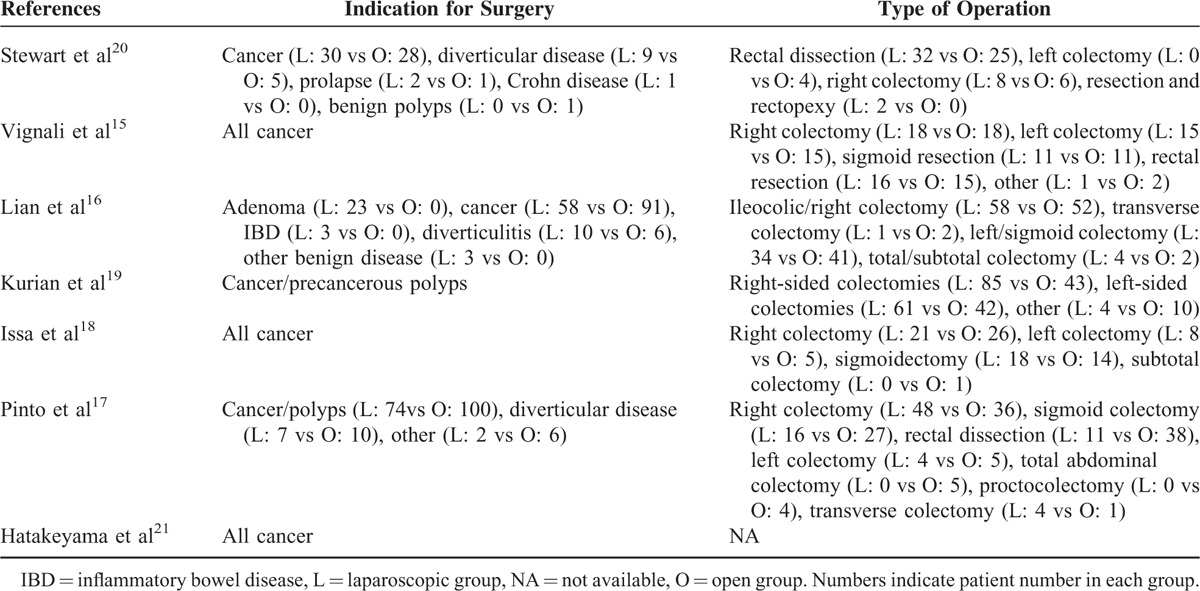
Surgery-related Details of Included Studies

**FIGURE 2:**
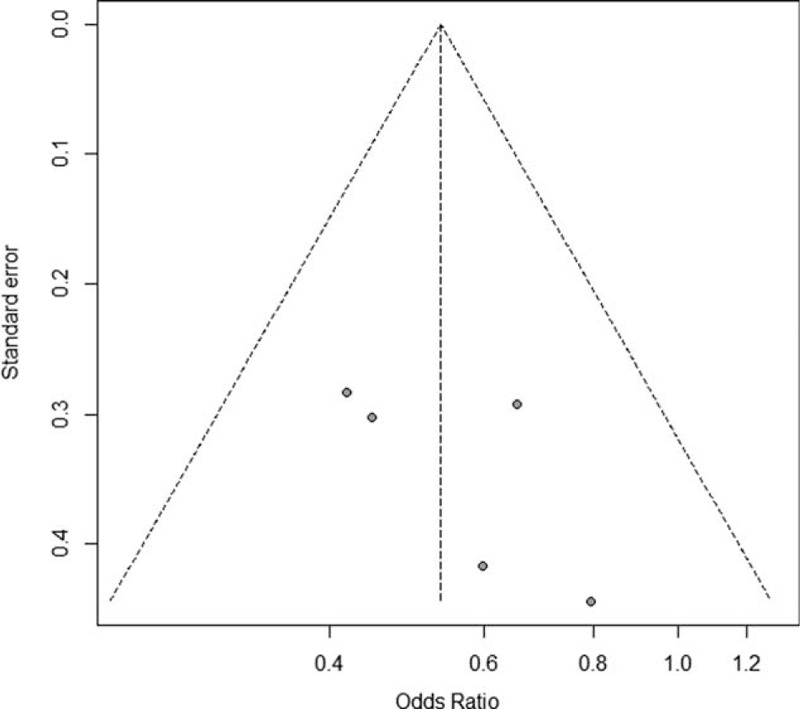
Funnel plot of included studies.

From the quality assessment based on the Newcastle–Ottawa Scale, all the studies included were of good to high quality (≥6 stars). As shown in Figure 2, the funnel plot revealed no publication bias of the included studies (*P* = 0.65). Eleven measurement indices comparing LC versus OC were concerned, which were listed in Table 3 after pooled analysis.

**TABLE 3:**
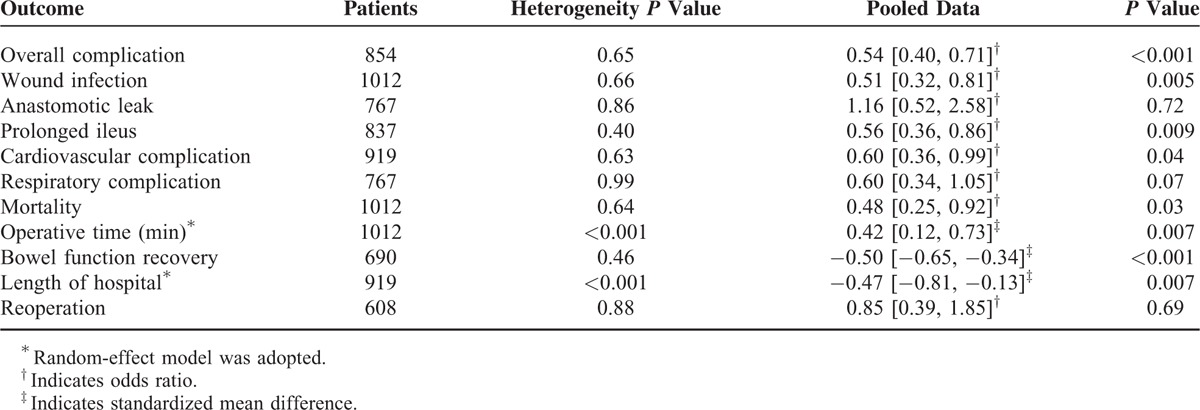
Meta-analysis of the Outcomes After Laparoscopic and Open Colectomy

### Safety Outcomes

#### Overall Complication

**FIGURE 3:**
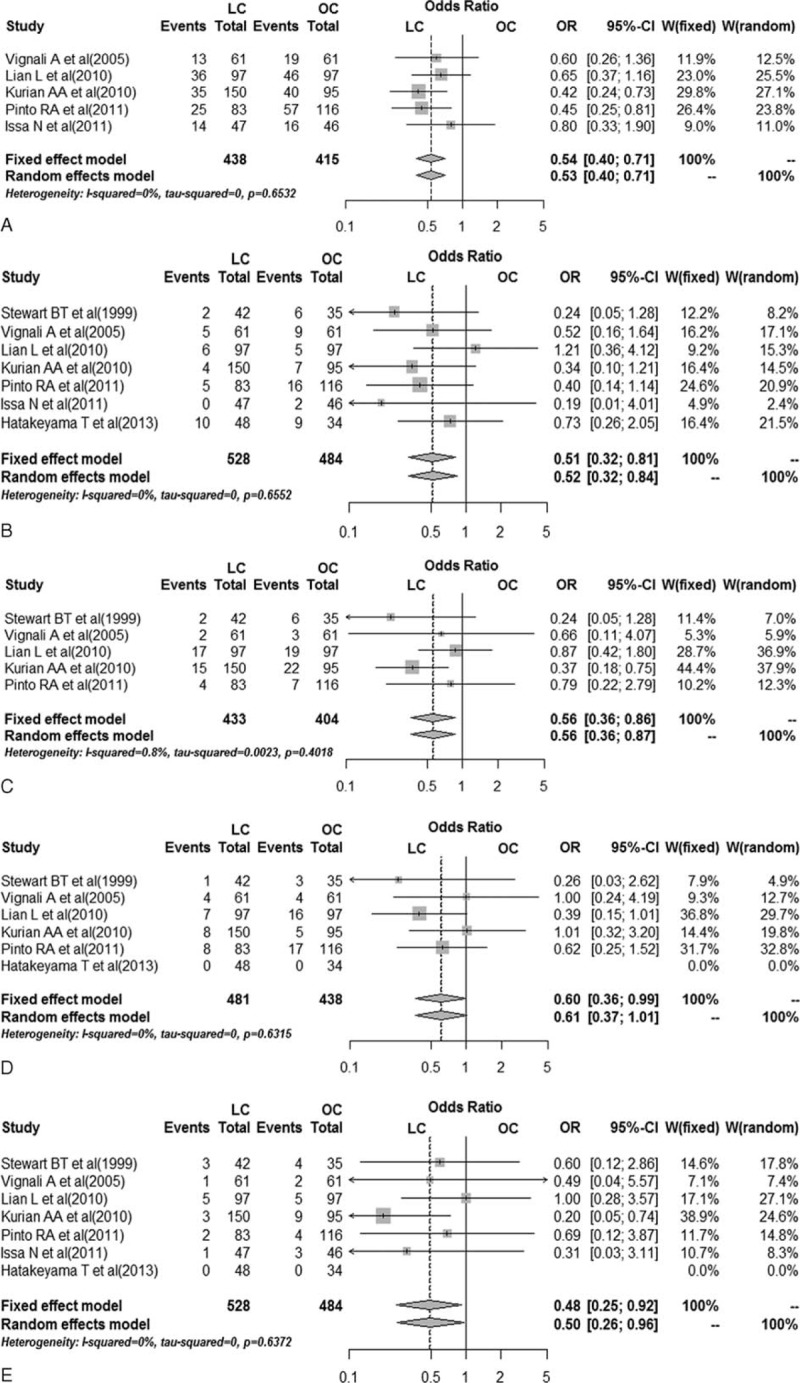
Pooled data on safety outcomes between laparoscopic and open colorectal resection in octogenarians. A, Forest plot of overall complication. B, Forest plot of wound infection. C, Forest plot of prolonged ileus. D, Forest plot of cardiovascular complication. E, Forest plot of mortality. LC = laparoscopic colorectal resection, OC = open colorectal resection, OR = odds ratio.

Five studies^15–19^ assessed occurrences of at least 1 complication (Figure 3A). The total complication rates were recorded. A pooled analysis combining the effects of 5 studies showed that the overall complication rate of LC group in the octogenarians was significantly lower than that of the OC group (28.1% vs 42.9%; OR 0.54, 95% CI 0.40–0.71, *P* < 0.001).

#### Wound Infection

Laparoscopic surgery in the octogenarians has a reportedly lower wound infection rate in all 7 studies^15–21^ (Figure 3B); however, the difference was statistically significant in only one of them.^20^ Overall, among the 1012 analyzed patients, LC was associated with a significantly lower incidence of wound infection rate when compared with OC (OR 0.51, 95% CI 0.32–0.81, *P* = 0.005).

#### Anastomotic Leak

Anastomotic leak was investigated in 6 studies,^15–18,20,21^ which showed no significant difference between the LC and the OC groups in octogenarians. After pooling the effects, the difference remained statistically insignificant (OR 1.16, 95% CI 0.52–2.58, *P* = 0.72).

#### Prolonged Ileus

The prolonged ileus rate was reported in 5 studies including 837 patients (Figure 3C).^15–17,20^ Only 1 study found statistical difference.^19^ After pooling the effects, LC was associated with a significantly lower incidence of prolonged ileus rate when compared with OC (OR 0.56, 95% CI 0.36–0.86, *P* = 0.009).

#### Cardiovascular Complication

Data on cardiovascular complication were reported in 6 studies^15–17,19–21^ including 919 patients, which showed lower cardiovascular complication rate in the laparoscopic surgery group (Figure 3D). Only 1 study found statistical difference.^20^ By pooling the data, it was found that LC was associated with lower cardiovascular complication rate (OR 0.60, 95% CI 0.36–0.99, *P* = 0.04).

#### Respiratory Complication

Pooled analysis of respiratory complication across 6 studies^15–18,20,21^ noted that there was no difference between the 2 treatment groups (OR 0.60, 95% CI 0.34–1.05, *P* = 0.07).

#### Mortality

Postoperative mortality here was defined as the death within 30 days of surgery. Pooled data from all 7 studies^15–21^ showed that LC was associated with a significantly lower mortality when compared with OC (OR 0.48, 95% CI 0.25–0.92, *P* = 0.03) (Figure 3E).

### Surgical Outcomes

#### Bowel Function Return

**FIGURE 4:**
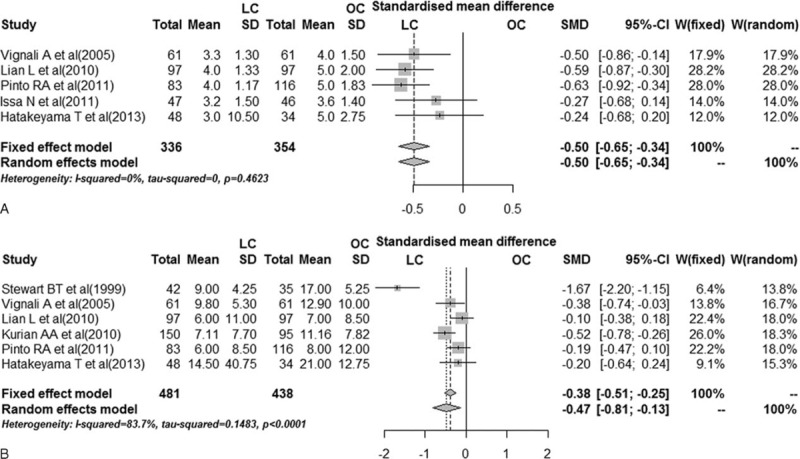
Pooled data on surgical outcomes between laparoscopic and open colorectal resection in octogenarians. A, Forest plot of bowel function return time. B, Forest plot of length of hospital stay. LC = laparoscopic colorectal resection, OC = open colorectal resection, SMD = standardized mean difference.

Data on bowel function return were available in 5 studies^15–18,21^ including 690 patients. The bowel function return was defined by oral intake initiated after the first flatus. The data were recorded starting on the morning after surgery for 24 h. Pooled analysis showed a shorter recovery time in the laparoscopic group (OR −0.50, 95% CI −0.65 to −0.34, *P* < 0.001) (Figure 4A).

#### Length of Hospital Stay

Issa et al^18^ reported postoperative hospital stay, whereas the other 6 studies concerned overall hospital stay. We conducted overall hospital stay by pooling the 6 studies,^15–17,19–21^ showing a favorable heterogeneity (*I*^2^ = 83.7%). The random-effect model was used, which suggested that laparoscopic approach was associated with a shorter hospital stay (SMD −0.47, 95% CI −0.81 to −0.13, *P* = 0.007) (Figure 4B).

#### Reoperation

Four studies^15–18^ including 608 patients reported the occurrence of reoperation. There was no significant difference found between the 2 groups in all the 4 studies. The pooled OR was comparable (OR 0.85, 95% CI 0.39–1.85, *P* = 0.70).

## DISCUSSION

This systematic review and meta-analysis of observational studies was performed to compare clinical outcomes between LC versus OC in octogenarians. The 7 included observational studies available so far suggest that laparoscopic approach was associated with lower rate of prolonged ileus, mortality, and overall complications, as well as quicker bowel function return and shorter length of hospital stay. The short-term outcomes of laparoscopic colorectal resection seem to be more favorable in octogenarians.

Over the past decade, laparoscopy colorectal resection is now accepted as not only feasible but also advantageous.^8,9,22^ Previous studies have shown that colorectal surgery in older patients is generally well tolerated as compared to that in younger patients.^10,23^ The surgical outcomes of LC in octogenarians have been previously studied with conflicting results. Compared with open surgery, laparoscopic colorectal resection demonstrated better short-term outcomes in several published randomized clinical trials.^5,7,8^ However, older patients with significant comorbidities were excluded in the majority of these clinical trials. Therefore, more data are needed to confirm the potential benefits of LC in the octogenarians.

The hemodynamic and ventilation changes observed in laparoscopic surgery were considered as concerning factors for the octogenarians.^24^ On the contrary, the minimal invasive nature of laparoscopy may minimize surgical stress onto the older patients. Possible benefits of laparoscopy in this age group remain unclear. Therefore, the adoption of laparoscopy in octogenarians has not been a common practice. This study aimed to compare the safety and surgical outcomes of LC with OC for the octogenarians.

The results of this meta-analysis suggest that laparoscopic resection is advantageous in most of the analyzed outcomes for colorectal diseases in octogenarians. Lower overall complication rate and less wound complication are shown as observed in other age groups.^9,22^ Although longer operation time and the Trendelenburg position are usually required during a laparoscopic procedure, fewer cardiovascular complication and similar respiratory complication suggest that the hemodynamic and ventilation change are well tolerated and laparoscopic technique could be safely used in older patients. This is in accordance with the study by Zhu et al^25^ which showed that with cautious perioperative monitoring, carbon dioxide pneumoperitoneum is safe for the older patients without significant adverse effects. The benefits brought by the less surgical operation and stress prevailed over the disadvantages in laparoscopy mentioned above in the octogenarians. Fielding et al^26^ reported that pulmonary complications are among the most significant problems in the postoperative period after colorectal resections, and cardiopulmonary complications after surgery in older patients are a major cause of postoperative morbidity and mortality. Lower overall complications perhaps contributed to the lower mortality between the 2 procedures in this study. About surgery-related complications, there were no differences in anastomotic leak and lower incidence of prolonged ileus. All complications mentioned above suggest that LC in the octogenarians is at least as safe as open surgery.

Length of hospital stay in octogenarians after LC has been shown to be shorter than that of the OC group. A newly published retrospective literature by White et al^27^ found similar results on postoperative complications and length of hospital stay of LC versus OC in octogenarians. However, necessary data were not provided in this study, so it was not included in this meta-analysis. We also found a quicker bowel function return for the laparoscopic group. These results are in accordance with results of several other published reports, indicating that patients in other age groups undergoing LC have quicker postoperative recovery when compared to OC.^9,28^ These findings suggest that the overall cost may be decreased when laparoscopic approach is used for octogenarians, which cannot be determined because of unavailability of the pertinent data.

The present study has inherent limitations. Firstly, all the included studies were retrospective design with selection bias. Larger sample and better-designed studies are needed to verify our conclusions. Secondly, the patient characteristics, surgical protocols, surgeon experience, and postoperative care regimens are not unified among the included studies and sometimes not available. For example, data were recorded by the median and range in some studies, but others used mean and standard deviation. Therefore, bias might not be obviated.

## CONCLUSIONS

Laparoscopic colorectal resection is as safe as open approach, and the short-term outcomes seem to be more favorable in octogenarians. However, clinical value of LC in octogenarians needed further evaluation with better-designed and long-term follow-up studies.
